# Preferences of Young Adults With First-Episode Psychosis for Receiving Specialized Mental Health Services Using Technology: A Survey Study

**DOI:** 10.2196/mental.4400

**Published:** 2015-05-20

**Authors:** Shalini Lal, Jennifer Dell'Elce, Natasha Tucci, Rebecca Fuhrer, Robyn Tamblyn, Ashok Malla

**Affiliations:** ^1^ School of Rehabilitation University of Montreal Montreal, QC Canada; ^2^ School of Physical and Occupational Therapy McGill University Montreal, QC Canada; ^3^ Institute of Psychiatry Psychology and Neuroscience King's College London London United Kingdom; ^4^ McGill University Department of Epidemiology, Biostatistics, and Occupational Health Montreal, QC Canada; ^5^ Department of Psychiatry McGill University Montreal, QC Canada

**Keywords:** eHealth, health services accessibility, medical informatics, mHealth, patient engagement, telemedicine

## Abstract

**Background:**

Despite the potential and interest of using technology for delivering specialized psychiatric services to young adults, surprisingly limited attention has been paid to systematically assess their perspectives in this regard. For example, limited knowledge exists on the extent to which young people receiving specialized services for a first-episode psychosis (FEP) are receptive to using new technologies as part of mental health care, and to which types of technology-enabled mental health interventions they are amenable to.

**Objective:**

The purpose of this study is to assess the interest of young adults with FEP in using technology to receive mental health information, services, and supports.

**Methods:**

This study uses a cross-sectional, descriptive survey design. A convenience sample of 67 participants between the ages of 18 and 35 were recruited from two specialized early intervention programs for psychosis. Interviewer-administered surveys were conducted between December 2013 and October 2014. Descriptive statistics are reported.

**Results:**

Among the 67 respondents who completed the survey, the majority (85%, 57/67) agreed or strongly agreed with YouTube as a platform for mental health-related services and supports. The top five technology-enabled services that participants were amenable to were (1) information on medication (96%, 64/67); (2) information on education, career, and employment (93%, 62/67); (3) decision-making tools pertaining to treatment and recovery (93%, 62/67); (4) reminders for appointments via text messaging (93%, 62/67); and (5) information about mental health, psychosis, and recovery in general (91%, 61/67). The top self-reported barriers to seeking mental health information online were lack of knowledge on how to perform an Internet search (31%, 21/67) and the way information is presented online (27%, 18/67). Two thirds (67%; 45/67) reported being comfortable in online settings, and almost half (48%; 32/67) reported a preference for mixed formats when viewing mental health information online (eg, text, video, visual graphics).

**Conclusions:**

Young people diagnosed with FEP express interest in using the Internet, social media, and mobile technologies for receiving mental health-related services. Increasing the awareness of young people in relation to various forms of technology-enabled mental health care warrants further attention. A consideration for future research is to obtain more in-depth knowledge on young people’s perspectives, which can help improve the design, development, and implementation of integrated technological health innovations within the delivery of specialized mental health care.

## Introduction

Internet and mobile technologies are increasingly being considered as a promising avenue to improve access and quality of mental health services [1-5]. This is particularly true for young people, given the omnipresence of online technologies in their daily lives [6-8]. Technology can be used to support and complement many areas of mental health service delivery including providing information, conducting screenings and assessments, monitoring symptoms and behaviors, delivering psychosocial interventions, and providing peer support [1]. Technology-enabled services offer a less intensive and arguably a more engaging format that is commensurate with the developmental culture of young people growing up in the 21st century [9]. However, there is a significant gap between the role that technologies such as the Internet, social media, and mobile devices play in young people’s lives and the role that these technologies have in the delivery of specialized psychiatric services to young people. For example, specialized early intervention (SEI) services for young people diagnosed with a first-episode psychosis (FEP) are predominantly based on models of care that are delivered in person.

As specialized psychiatric services begin to consider various forms of technology to augment, complement, or extend the reach of care for young people, it is important to obtain their interests and perspectives in this regard. For example, information is needed on young people’s preferences for receiving technology-enabled mental health care, and which types of services they are amenable to (eg, online peer support, text reminders for medication and appointments, online counseling). This knowledge can guide the development of Internet-based and mobile innovations that are engaging, useful, and patient centered. As such, the aim of this paper is to describe the methods and results of a survey we conducted to assess the preferences of young adults with FEP on using technology for receiving mental health services, information, and supports.

## Methods

### Recruitment

Using a cross-sectional, descriptive survey design, a total of 67 participants were recruited from two SEI programs for psychosis: Prevention and Early Intervention Program for Psychoses-Montréal and Prevention and Early Intervention Program for Psychoses-McGill University Health Centre. Before being admitted to these services, the majority of patients received less than 1 month of medication for their FEP. Ethical approval was received from the Institutional Review Board of McGill University’s Faculty of Medicine, and all participants provided informed consent.

Participants were eligible if they met the following criteria: between 18 and 35 years of age, diagnosed with an affective or nonaffective psychotic disorder, within 5 years of treatment, clinically stable, and able to communicate in English or French. Participants were excluded if they had no contact with clinical staff for over 3 months, were hospitalized at the time of recruitment, were unable to concentrate or attend to a conversation, or were intoxicated at the time of recruitment. Recruitment occurred between December 2013 and October 2014.

### Measures

Data collection involved an interviewer-administered survey (20-30 minutes in duration), developed iteratively over a 3-month period with feedback from young adult patients and family representatives, service providers, and the research team. The survey consisted of quantitative questions to assess the following data: (1) demographics; (2) access and use of technology and social media; and (3) preferences regarding the use of technology for various types of mental health services, information, and supports. In this paper, items 1 and 3 were included. Examples of questions from the survey are provided in Multimedia Appendix 1. Responses were numerically coded in an SPSS database (version 20; SPSS Inc, Chicago, IL, USA) and descriptive statistics (ie, mean, standard deviation, frequencies) are reported in the following sections.

## Results

### Participants

Of the 76 individuals approached, 67 provided consent and completed the survey. The mean age of the participants was 25.6 (standard deviation = 5.1), and 76% were men (51/67). Table 1 presents the demographic details of the participants.

**Table 1:** Demographic characteristics (n=67).

Characteristics		n	%
**Sex (male/female)**			
	Male	51	76.1
**Language of survey (English/French)**			
	English	33	49.3
**Length of treatment**			
	>12 months	39	58.2
	6-12 months	17	25.4
	<6 months	11	16.4
**Race** ^a^			
	White	43	64.2
	Asian	10	14.9
	Black	5	7.5
	Latin American	5	7.5
	Arab	1	1.5
	Mixed	3	4.5
**Education**			
	High school complete	23	34.3
	High school incomplete	16	23.9
	College complete	15	22.4
	Undergraduate studies complete	10	14.9
	Graduate studies complete	3	4.5
**Employment status**			
	Not employed/looking for work	42^b^	62.7
	Full time	17	25.4
	Part-time	8	11.9
**Education status**			
	Not in school/not student	49^c^	73.1
	Part-time	10	14.9
	Full time	8	11.9
**Living situation**			
	With family/guardian	42	62.7
	Alone	10	14.9
	With roommates	7	10.4
	With spouse	6	9.0
	Supported housing	2	3.0
**Annual income (CAD$)**			
	<14,999	49	73.1
	15,000-29,999	7	10.4
	30,000-49,999	9	13.4
	>50,000	2	3.0
**Children (Yes/No)**			
	No	60	89.6

^a^Race categories were adapted from Statistics Canada race breakdown.

^b^Eight of the 42 are full-time students and four others are on medical leave.

^c^Two of the 49 are on medical leave from school.

### Social Media and Mental Health Services

In descending order, participants agreed or strongly agreed with the idea of using the following social media sites as platforms to receive mental health information and supports: YouTube (85%, 57/67), Facebook (58%, 39/67), Skype (40%, 27/67), and Twitter (39%, 26/67).

### Technology and Various Types of Mental Health-Related Services

As illustrated in Table 2, in descending order, the participants were amenable to using technology for the following top 10 types of services:(1) information on medication and side effects; (2) information and support related to education, career, and employment; (3) decision-making tools regarding treatment and recovery; (4) reminders for appointments through text messaging; (5) information about mental health, psychosis, and recovery in general; (6) information about physical health; (7) contact with mental health care providers; (8) scheduling appointments; (9) information about program events; and (10) education on coping skills. More than half (66%, 44/67) agreed or strongly agreed with using technology to facilitate social contact with other young people receiving services for FEP, and a little more than half of the participants (52%, 35/67) agreed or strongly agreed with receiving counseling or therapy online.

**Table 2:** Types of technology-enabled services (n=67).

Service		Strongly agree/agree n (%)	Strongly disagree/disagree n (%)	Undecided n (%)
**Reminders for appointments**				
	By text message	62 (92.5)	3 (4.5)	2 (3.0)
	By email	53 (79.1)	9 (13.4)	5 (7.5)
	By applications	49 (73.1)	9 (13.4)	9 (13.4)
**Reminders for medication**				
	By text message	45 (67.1)	16 (23.9)	6 (9.0)
	By email	38 (56.7)	22 (32.8)	7 (10.4)
	By applications	42 (62.7)	15 (22.4)	10 (14.9)
Information on medications and side effects		64 (95.5)	1 (1.5)	2 (3.0)
Information/support related to education, career, and employment		62 (92.5)	3 (4.5)	2 (3.0)
Tools to enable decision making regarding treatment and recovery		62 (92.5)	3 (4.5)	2 (3.0)
Information on mental health, psychosis, and recovery		61 (91.0)	3 (4.5)	3 (4.5)
Information on physical health		61 (91.0)	4 (6.0)	2 (3.0)
Contact with treatment team		57 (85.1)	6 (9.0)	4 (6.0)
Appointment scheduling		56 (83.6)	7 (10.4)	4 (6.0)
Information on program events		56 (83.6)	7 (10.4)	4 (6.0)
Education on coping skills		56 (83.6)	7 (10.4)	4 (6.0)
Online social contact between clients in the program and clients from similar programs		44 (65.7)	13 (19.4)	10 (14.9)
Counseling/therapy services		35 (52.2)	18 (26.9)	14 (20.9)

### Barriers to Accessing Mental Health Support and Information Online

Table 3 describes the barriers that participants (n=67) reported in accessing mental health information and support online. In descending order, the top five barriers were (1) lack of knowledge on how to perform an Internet search (31%, 21/67), (2) the way in which information is presented online (27%, 18/67), (3) no interest or need (22%, 15/67), (4) lack of time (19%, 13/67), and (5) cost of Internet access (19%, 13/67). A little less than a third (30%; 20/67) reported “no barriers” to accessing mental health information and support online.

**Table 3:** Barriers to accessing mental health information and support online (n=67).

Barriers	n	%
Lack of knowledge on how to search Internet	21	31.3
No barriers	20	29.9
Way the information is presented online	18	26.9
Not interested/no need	15	22.4
Lack of time	13	19.4
Cost of Internet access	13	19.4
Lack of skills using technological devices	12	17.9
Fear or discomfort with technological devices	11	16.4
Lack of access to a device	9	13.4
Lack of access to Internet	5	7.5
Due to disability	4	6.0
Other^a^	2	3.0

^a^Other includes too much information, and knowing what information is good and bad.

### Preferred Formats for Receiving Mental Health Information Online

**Figure 1:**
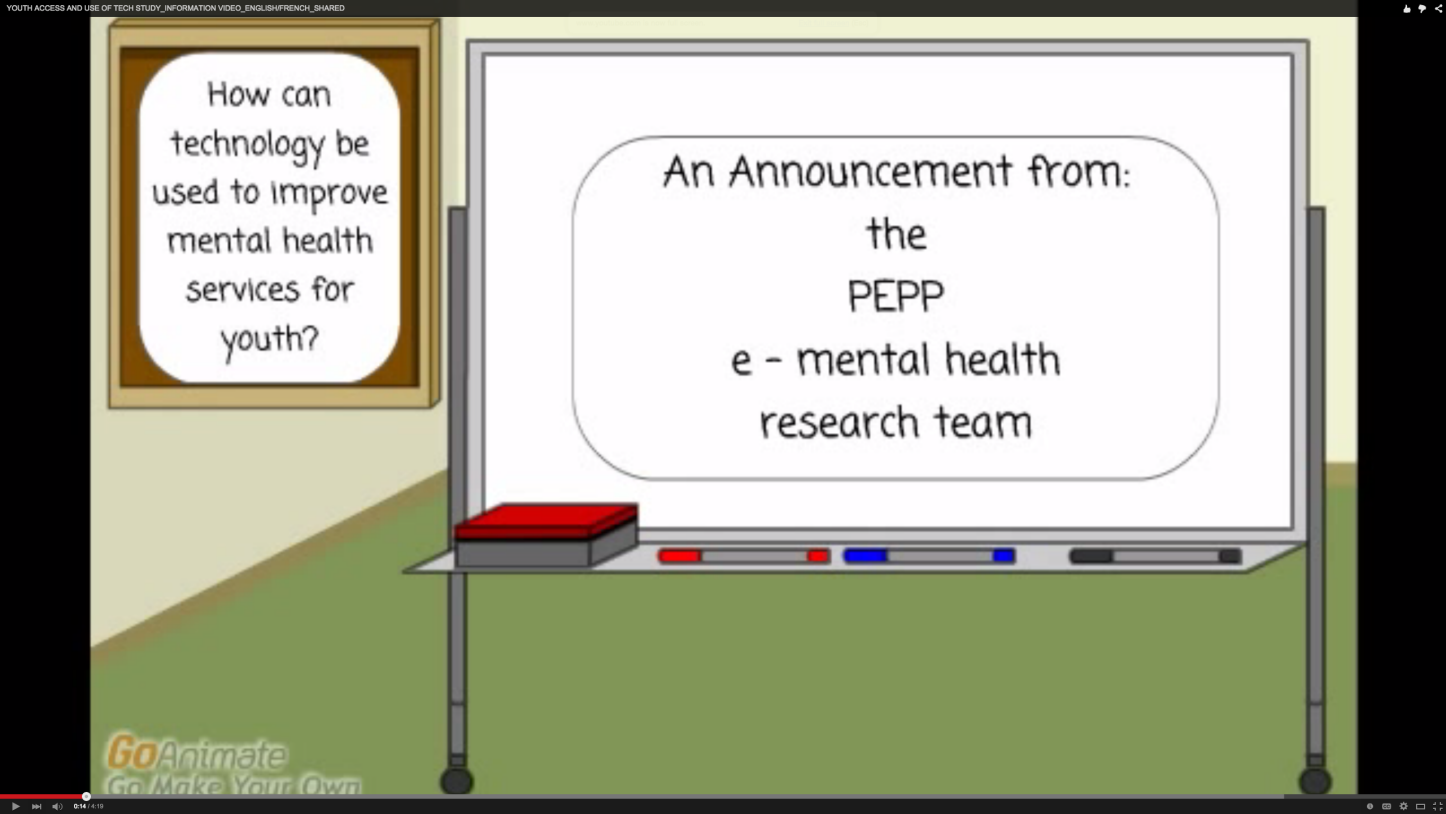
Screenshot from information video about the study.

Table 4 describes preferences for receiving mental health information online. Almost half of the participants (48%, 32/67) preferred mixed formats including a combination of text, video, graphics, and audio. Almost one third (31%, 21/67) preferred text as the method for receiving information, and 15% (10/67) preferred video (see Figure 1 for screenshot).

**Table 4:** Preferred formats for mental health information online (n=67).

Format	n	%
Mixed formats	32	47.8
Text	21	31.3
Video	10	14.9
Graphics	2	3.0
Audio	1	1.5
Other^a^	1	1.5

^a^The response indicated “no preference.”

### Comfort Levels in Different Online and Offline Social Environments

When asked about the level of comfort in different types of social environments, the following portion of participants reported feeling comfortable or very comfortable in online settings, over the phone, and in-person group settings: 67% (45/67), 66% (44/67), and 67% (45/67), respectively, whereas 81% of the sample (54/67) expressed being comfortable or very comfortable with one-to-one, in-person interactions.

## Discussion

### Principal Findings

To our knowledge, this study is the first to systematically assess preferences of young people with FEP for using various technologies to receive mental health care. We found that the majority of participants strongly agreed with using social media such as YouTube and Facebook as a platform for mental health-related services and supports. The majority of the participants also agreed with the idea of using technology for receiving information on topics such as medication, education, career, employment, mental health, psychosis, and recovery; as decision-making tools; and as reminders for appointments. Lack of knowledge on how to perform an Internet search and the way information is presented online were reported as barriers to seeking mental health information. Almost half of the participants reported a preference for mixed formats when viewing mental health information online (eg, text, video, visual graphics). It is also noteworthy that one third of participants reported lack of comfort in online settings.

YouTube and Facebook are the two most popular social media sites with young people in North America [10,11]. Social media offer young people a forum for asking questions, receiving feedback, sharing personal stories, and receiving information about clinical services and related events. However, it is important to note that there are many questions in terms of governance, ethics, professionalism, privacy, and confidentiality pertaining to the use of social media in the health care sector that remain unanswered [12,13]. Future research that includes the subjective perspectives of young people on these factors is warranted.

Given the extent to which young people are increasingly socialized to technology at a very young age, it is surprising that approximately one third of the sample reported lack of comfort in online environments. This may be related to privacy concerns, symptoms, or lack of skill level and confidence with technology; however, further research is needed to understand the factors contributing to this lack of comfort. Such knowledge can provide insight into the need for raising the literacy, confidence, and skills of young people in accessing mental health information, services, and supports online.

About one third disagreed, strongly disagreed, or were undecided regarding receiving reminders for taking medication through texting or email, which may be related to negative attitudes and perceptions about medication. Moreover, approximately half of the sample agreed with using technology to connect with peers or receive counseling. These findings can be better understood through qualitative research methods.

### Limitations

The findings of this study are based on young adults between 20 and 30 years of age receiving services for an FEP in Quebec, Canada, and may not be generalizable to youth elsewhere. The majority of the sample population was men and although FEP incidence rates are approximately two times as high in male participants compared with female participants [14], our sample is still gender biased. It is important for future research to consider gender-related preferences in relation to receiving technology-enabled mental health care. The findings are also limited by the fact that the study is based on self-reported data. Given the small sample size, the results should be interpreted with caution. Future research with a larger sample that can assess how sociodemographic factors (eg, gender, economic status, ethnicity, and education) influence preferences in relation to using technology for various types of services and supports is warranted.

### Conclusions

Our study provides preliminary indications that young adults with FEP are interested in using the Internet, social media, and mobile technologies for receiving specialized services. Young people with FEP are in an ideal position to contribute to how mental health information, services, and supports can best be translated into online formats. These results will help inform the development of a website tailored to the needs of young people receiving services for an FEP at the recruitment sites. Further research, through qualitative methods, is warranted on patient perspectives regarding how social media and other types of technologies can be used to enhance service delivery and the issues of confidentiality, security, ethics, and professionalism in this regard.

## Appendix

Multimedia Appendix 1 Examples of survey questions.

## Abbreviations

FEP: first-episode psychosis
SEI: specialized early intervention
