# Supplementary material for: Preferences of Young Adults With First-Episode Psychosis for Receiving Specialized Mental Health Services Using Technology: A Survey Study
Source: JMIR Ment Health. 2015 May 20;2(2):e18. doi: 10.2196/mental.4400 (PMC4607389; doi:10.2196/mental.4400)
Supplement: Multimedia Appendix 1 [file mental_v2i2e18_app1.pdf]

Survey Examples of Questions

1. *To what extent do you agree that the following social media can be used to receive education and support related to mental health topics?*

Check one box per topic	Strongly agree	Agree	Undecided	Disagree	Strongly disagree
**YouTube**					
**Facebook**					
**Myspace**					
**Twitter**					
**Skype**					
**Other:________________**					

2. *To what extent do you agree that PEPP should provide the following services with technology?*

**Reminders for appointments by**	**By text/SMS**	☐Strongly agree ☐Agree ☐Undecided ☐Disagree ☐Strongly disagree
	**By email**	☐Strongly agree ☐Agree ☐Undecided ☐Disagree ☐Strongly disagree
	**By use of an app**	☐Strongly agree ☐Agree ☐Undecided ☐Disagree ☐Strongly disagree
**Reminders for medications**	**By text/SMS**	☐Strongly agree ☐Agree ☐Undecided ☐Disagree ☐Strongly disagree
	**By email**	☐Strongly agree ☐Agree ☐Undecided ☐Disagree ☐Strongly disagree
	**By use of an app**	☐Strongly agree ☐Agree ☐Undecided ☐Disagree ☐Strongly disagree
**Education on coping skills**		☐Strongly agree ☐Agree ☐Undecided ☐Disagree ☐Strongly disagree
**Information on medication and side effects**		☐Strongly agree ☐Agree ☐Undecided ☐Disagree ☐Strongly disagree
**Education on mental health, psychosis, recovery**		☐Strongly agree ☐Agree ☐Undecided ☐Disagree ☐Strongly disagree
**Information on physical health**		☐Strongly agree ☐Agree ☐Undecided ☐Disagree ☐Strongly disagree
**Information and/or support related to education, career planning, and employment**		☐Strongly agree ☐Agree ☐Undecided ☐Disagree ☐Strongly disagree
**Scheduling appointments online**		☐Strongly agree ☐Agree ☐Undecided ☐Disagree ☐Strongly disagree
**Maintaining contact with PEPP-health care providers online**		☐Strongly agree ☐Agree ☐Undecided ☐Disagree ☐Strongly disagree
**Receiving counseling/therapy online from PEPP-health care providers**		☐Strongly agree ☐Agree ☐Undecided ☐Disagree ☐Strongly disagree

**Using social media to be in contact with other youth at PEPP or in programs similar to PEPP**	□Strongly agree □Agree □Undecided □Disagree □Strongly disagree
**Receiving information on upcoming events at PEPP (eg, group activities, special events)**	□Strongly agree □Agree □Undecided □Disagree □Strongly disagree
**Tools that help you to make decisions related to treatment and recovery (eg, taking medication, going back to work, and so forth)**	□Strongly agree □Agree □Undecided □Disagree □Strongly disagree
**Other:________________________________ ___________**	□Strongly agree □Agree □Undecided □Disagree □Strongly disagree

3. *Which of the following barriers would prevent you from mental health information and support online? Check all that apply.*

**Access to device**	
Cost of Internet access	
Access to Internet	
Lack of skills/training with technology	
Lack of knowledge about where the information is on the Internet	
The way information is offered	
Fear or discomfort with technology	
No need/Not interested	
Not enough time	
Disability (please specify): _____________	
I perceive no barriers	
Other: ____________	

4. *Which of the following formats do you most prefer for receiving mental health information online?*

**Text**	**Visual graphics**	**Video**	**Audio recording**	**Mix**	**Other**

If you checked “other,” please describe: ____

5. *How comfortable are you meeting new people in the following situations?*

	Very comfortable	Comfortable	Not very comfortable	Not comfortable at all
**Online**				
**By phone**				
**Face-to-face individually**				
**Face-to-face in a group**				
